# Testing Hopkins’ Bioclimatic Law with PhenoCam data

**DOI:** 10.1002/aps3.1228

**Published:** 2019-03-18

**Authors:** Andrew D. Richardson, Koen Hufkens, Xiaolu Li, Toby R. Ault

**Affiliations:** ^1^ School of Informatics, Computing, and Cyber Systems Northern Arizona University Flagstaff Arizona 86011 USA; ^2^ Center for Ecosystem Science and Society Northern Arizona University Flagstaff Arizona 86011 USA; ^3^ Department of Applied Ecology and Environmental Biology Ghent University Ghent Belgium; ^4^ INRA UMR ISPA Villenave d'Ornon France; ^5^ Department of Earth and Atmospheric Sciences Cornell University Ithaca New York 14853 USA

**Keywords:** Bioclimatic Law, digital repeat photography, green chromatic coordinate, PhenoCam, phenology, temperature sensitivity

## Abstract

**Premise of the Study:**

We investigated the spatial and temporal patterns of vegetation phenology with phenometrics derived from PhenoCam imagery. Specifically, we evaluated the Bioclimatic Law proposed by Hopkins, which relates phenological transitions to latitude, longitude, and elevation.

**Methods:**

“Green‐up” and “green‐down” dates—representing the start and end of the annual cycles of vegetation activity—were estimated from measures of canopy greenness calculated from digital repeat photography. We used data from 65 deciduous broadleaf (DB) forest sites, 18 evergreen needleleaf (EN) forest sites, and 21 grassland (GR) sites.

**Results:**

DB green‐up dates were well correlated with mean annual temperature and varied along spatial gradients consistent with the Bioclimatic Law. Interannual variation in DB phenology was most strongly associated with temperature anomalies during a relatively narrow window of time. EN phenology was not well correlated with either climatic factors or spatial gradients, but similar to DB phenology, interannual variation was most closely associated with temperature anomalies. For GR sites, mean annual precipitation explained most of the spatial variation in the duration of vegetation activity, whereas both temperature and precipitation anomalies explained interannual variation in phenology.

**Discussion:**

PhenoCam data provide an objective and consistent means by which spatial and temporal patterns in vegetation phenology can be investigated.

Understanding the spatial and temporal patterns of vegetation phenology, and the underlying drivers of those patterns, is important for predicting the phenological impacts of climate change (Richardson et al., 2013a). Can temporal variation be predicted from spatial variation, or vice versa? The realism of space‐for‐time assumptions has implications for accurately projecting phenological shifts under novel climate regimes (Mäkelä, 2013).

There is a long history of research into spatial patterns in ecology. During the late 19th and early 20th centuries, American entomologist Andrew Delmar Hopkins developed the Bioclimatic Law, which hypothesized that phenological events were shifted by four days for 1° latitude north, 5° longitude west, and 400 ft (120 m) of elevation increase (Hopkins, 1900, 1920a, b). The Bioclimatic Law was established through observation of the Hessian fly (*Mayetiola destructor*), an insect pest of many cereal crops. Hopkins proposed his rule of thumb to predict the end of the fly's fall swarm. After this date, wheat could safely be sown without risk of attack, while at the same time planting time would be optimized to ensure the best crop the following year (Hopkins, 1900). Hopkins later suggested that the Bioclimatic Law applied generally to many phenological events in plants, insects, and animals (Hopkins, 1920a).

Hopkins published extensively on his Bioclimatic Law (Hopkins, 1919) and viewed his monograph on the topic, *Bioclimatics: A Science of Life and Climate Relations* (Hopkins, 1938), as the culmination of his life's work. Although not universally accepted—Furniss (2010) wrote that the Law's “depiction of the relationships involved is too rigidly defined to fit the diversity that I have come to know in nature”—Hopkins’ work continues to be well cited almost a century later (Fitzjarrald et al., 2001; Richardson et al., 2006; Zhao et al., 2013; Dai et al., 2014; Klosterman et al., 2014; Liang, 2016; Vitasse et al., 2018).

Lacking extensive ground observations with which to test the Bioclimatic Law, in 1919 Hopkins and three colleagues embarked on a rail journey of over 20,000 miles across the eastern half of the United States to collect springtime phenological observations (Hopkins, 1920a, b). In the century since, more systematic data sets—including extensive citizen science observations (Hopp, 1976; Vitasse et al., 2018) and land surface phenological metrics from satellite remote sensing (Hudson Dunn and de Beurs, 2011; Zhao et al., 2013)—have been analyzed in the context of the Law.

Increasingly over the past decade, digital repeat photography has been used by researchers to track vegetation phenology in all manner of ecosystem types, from tropics to tundra (Richardson et al., 2007, 2013b; Ide and Oguma, 2010; Sonnentag et al., 2012; Alberton et al., 2014; Nasahara and Nagai, 2015; Anderson et al., 2016). PhenoCam, a collaborative network targeting the phenology of North American ecosystems (Richardson et al., 2018a), is just one of several such efforts worldwide. Following the standard PhenoCam protocol, images are masked for processing so that analysis can be conducted on a single vegetation type, thereby minimizing the mixed‐pixel challenge of moderate‐resolution satellite imagery. At the same time, PhenoCam data are well suited to canopy‐level integration across multiple taxa of the same plant functional type, thereby increasing representativeness. Finally, phenometrics derived from camera imagery are both objective and consistent in interpretation—i.e. characterizing the timing of “green up” and “green down”—across sites and vegetation types. Thus, this approach addresses some of the major limitations of both observer‐ and satellite‐based phenological data (Richardson et al., 2013b).

Here, we use the PhenoCam Dataset version 1.0 (Richardson et al., 2018a) to examine the factors associated with spatial and interannual variation in the timing of the “greenness rising” and “greenness falling” phenological transitions across three key vegetation types: deciduous broadleaf forest, evergreen needleleaf forest, and grassland. This analysis seeks to address whether phenological variation across sites is better explained by climate statistics (mean annual temperature and precipitation) or site location (i.e., the Bioclimatic Law: site latitude, longitude, and elevation). We extend the same analysis to estimated “green season length” (GSL), a proxy for the annual period of vegetation activity. Complementing these analyses, we used exploratory data analysis (stepwise regression) to link phenological anomalies (e.g., early vs. late timing in a given year) to variation in air temperature and precipitation during the weeks leading up to, and surrounding, the mean transition date at each site, to investigate whether the interannual correlates of phenological variation match with the spatial correlates.

## METHODS

Our analysis leverages data products derived from PhenoCam digital repeat photography, and observed and modeled lilac (*Syringa* L.) phenology, together with gridded climatological and meteorological data sets. We describe these data first, and then summarize the methods of statistical analysis.

### PhenoCam data

We used the PhenoCam Dataset version 1.0 (Richardson et al., 2017), which is publicly available under the Creative Commons CC0 Public Domain Dedication. We used all camera sites (Appendix S1) located in the continental United States or Canada (west of −30° longitude, north of 25° latitude) and featuring deciduous broadleaf forest (DB), evergreen needleleaf forest (EN), or grassland (GR) vegetation.

Image and data processing routines are fully described by Richardson et al. (2018a), but are summarized here. We defined a region of interest (ROI), corresponding to the masked area from which color information would be extracted for a given vegetation type. Images were read sequentially, and the mean pixel value (digital number [DN]) was determined across the ROI mask for each of the red, green, and blue (RGB) color channels. This yielded an “RGB DN triplet” (*R*_DN_, *G*_DN_, *B*_DN_) for each image. From the RGB triplet we calculated the Green Chromatic Coordinate (*G*_CC_; Eq. [Link]) (Sonnentag et al., 2012; Richardson et al., 2013b).

$$G_{\mathrm{cc}}=\frac{G_{\mathrm{DN}}}{R_{\mathrm{DN}}+G_{\mathrm{DN}}+B_{\mathrm{DN}}}$$

We calculated the 90th percentile value of *G*_CC_ across all images recorded over a three‐day moving window. This method has been shown to be effective for minimizing variation due to weather and illumination geometry (Sonnentag et al., 2012). We used a spline‐based method for outlier detection and removal (Richardson et al., 2018a). Briefly, the optimal flexibility of the spline was determined using a version of Akaike's Information Criterion to balance goodness‐of‐fit against model complexity (Hurvich et al., 1998). The spline was re‐fit and used to extract phenological transition dates from the *G*_CC_ time series. We applied the pruned exact linear time (PELT) method (Killick et al., 2012) to parse the “greenness rising” and “greenness falling” phenophases from each spline. We defined transition dates as the day of year the spline reached 10% of the seasonal amplitude for each phenophase. Transition date uncertainties were estimated based on the uncertainty around the smoothing spline (95% confidence interval). The mean uncertainty was about ±5 days, with a median of ±2.5 days.

### Observed and modeled lilac phenology

To further investigate phenological patterns across sites, we used (1) an expanded lilac (*Syringa*) data set (Schwartz and Caprio, 2003), with broader spatial and temporal coverage than that previously analyzed by Hopp (1976), and (2) USA National Phenology Network (2018) estimates of lilac and honeysuckle phenology generated using the “extended Spring Index” (SI‐x) models (Schwartz et al., 2006) driven by gridded daily weather data from the PRISM Climate Group (Daly et al., 2008).

The lilac data set covers 1956–2003 for 1126 locations in the United States and Canada, with observations for both a cultivar, *S*. ×*chinensis* (as in Hopp, 1976) (195 sites, 16 ± 8 y [mean ± 1 SD] of “first leaf” observations and 15 ± 8 y of “first bloom” observations per site), as well as common lilac, *S. vulgaris* L. (931 sites, 6 ± 6 y of “first leaf” observations and 12 ± 7 y of “first bloom” observations per site). An advantage of the cultivar is that it is cloned, and hence genetic variation among individuals is minimized. Thus, for *S*. ×*chinensis* the spatial patterns should be driven by environmental variation.

The SI‐x models integrate winter and spring weather in a biologically meaningful way, and thus spatial patterns in model predictions should also represent environmental variation (Ault et al., 2011). However, whereas lilac observations were conducted across a non‐random sample of sites distributed across the western (*S. vulgaris*) and eastern (*S*. ×*chinensis*) United States, we conducted wall‐to‐wall SI‐x analyses, eliminating potential sampling biases. We then restricted our SI‐x analysis just to those grid cells in which PhenoCams were located, in order to simulate the potentially inadequate sampling of that data set. The latter analysis was done first using mean SI‐x predictions over 2000–2015 and then using mean SI‐x predictions only for the years for which corresponding green‐up estimates were available.

### Climatological and meteorological data

For our analysis of spatial patterns in vegetation phenology across sites, we used WorldClim (Hijmans et al., 2005) (http://worldclim.org/) estimates of mean annual temperature (°C) and mean annual precipitation (mm), reported at a spatial resolution of approximately 1 km^2^. For our analysis of interannual variation in phenology within sites, we used daily temperature and precipitation data from Daymet (Thornton et al., 1997, 2016) (http://daymet.ornl.gov/), reported at a spatial resolution of 1 km for continental North America south of 52°N latitude. Daymet data were unavailable for two high‐latitude PhenoCam sites.

### Statistical analysis

Regression analyses were conducted in SAS version 9.1 using *proc reg* and *proc glm* (SAS Institute Inc., 2008). For analyses of spatial patterns in phenology using the climate statistics (phenology = *f*[mean annual temperature, mean annual precipitation]) and site location (phenology = *f*[latitude, longitude, elevation]) models, we first calculated the mean phenological transition date across all years of observations for each site. We then weighted sites according to the number of observations per site (*n*). This accounts for the non‐constant variance across sites (heteroskedasticity; i.e., the standard error of each mean scales with $1/\sqrt{n}$): weighted least squares thus yields the best linear unbiased estimators (BLUE) of the parameters (SAS Institute Inc., 2008). (But note that the parameter estimates were not substantially different, and our interpretation of the results was unchanged, when ordinary least squares was used instead.) We used a significance level of α = 0.05.

For analysis of interannual variation within sites, we calculated transition date anomalies from the site‐level mean for each phenometric. We excluded individual transition dates when the transition date uncertainty (95% confidence interval) was >20 d (<4% of all observations). We also calculated anomalies in mean air temperature and total precipitation, for six 20‐day moving windows, from 90 days before the site‐level mean transition date to 30 days after the site‐level mean transition date. We use the notation $sT_{x}^{y}$ to represent the phenological sensitivity to mean temperature (in days per 1°C), calculated over the 20‐day period beginning on day *x* and ending on day *y* (= *x* + 19) relative to the mean transition date, where negative values correspond to dates before the mean transition date. The phenological sensitivity to total precipitation (in days per 100 mm) is likewise denoted $sP_{x}^{y}$.

We used stepwise regression (Neter et al., 1996) in *proc reg* (SAS Institute Inc., 2008) to identify the periods of phenological sensitivity to variation in weather, i.e., where $sT_{x}^{y}$ or $sP_{x}^{y}$ were significantly different from zero. The stepwise method is a variation on standard forward‐selection techniques, in that at each step candidate variables are added, one at a time, to the model if they improve model fit (*F*‐test, SLENTRY ≤ 0.10). Variables in the model are subsequently removed if, after incorporation of other variables, they are no longer significant (*F*‐test, SLSTAY ≤ 0.10). The process is considered complete when no variables not in the model meet the SLENTRY criterion, and every variable in the model meets the SLSTAY criterion. A minimum of 2 y of data was required for sites to be included in this analysis. Transition dates were weighted by the squared reciprocal of the estimated uncertainty, again to yield BLUE parameters (but similar results were obtained using ordinary least squares).

Although there were more than 350 site‐years of data for this analysis for DB sites, there were only ≈50 site‐years for EN sites, and ≈80 site‐years for GR sites. However, in all cases there were at least 10 times as many data points as fit parameters in the model. Nevertheless, we view this as exploratory data analysis, rather than rigorous hypothesis testing, and these results should be interpreted in that context.

## RESULTS

### Geographic and eco‐climatic distribution of sites

We used data from 93 camera sites (Appendix S1)—in total more than 560 site‐years of imagery—spanning 2000 to 2015. The average image time series was over 6 y in length. The shortest image time series was eight months long, while the longest was almost 16 y. Thirteen camera sites had more than a decade of imagery. The data set was heavily weighted toward more recent years, with 60% of all data from 2012–2015 (approximately equal representation by year), and only 12% pre‐2008.

**Figure 1:**
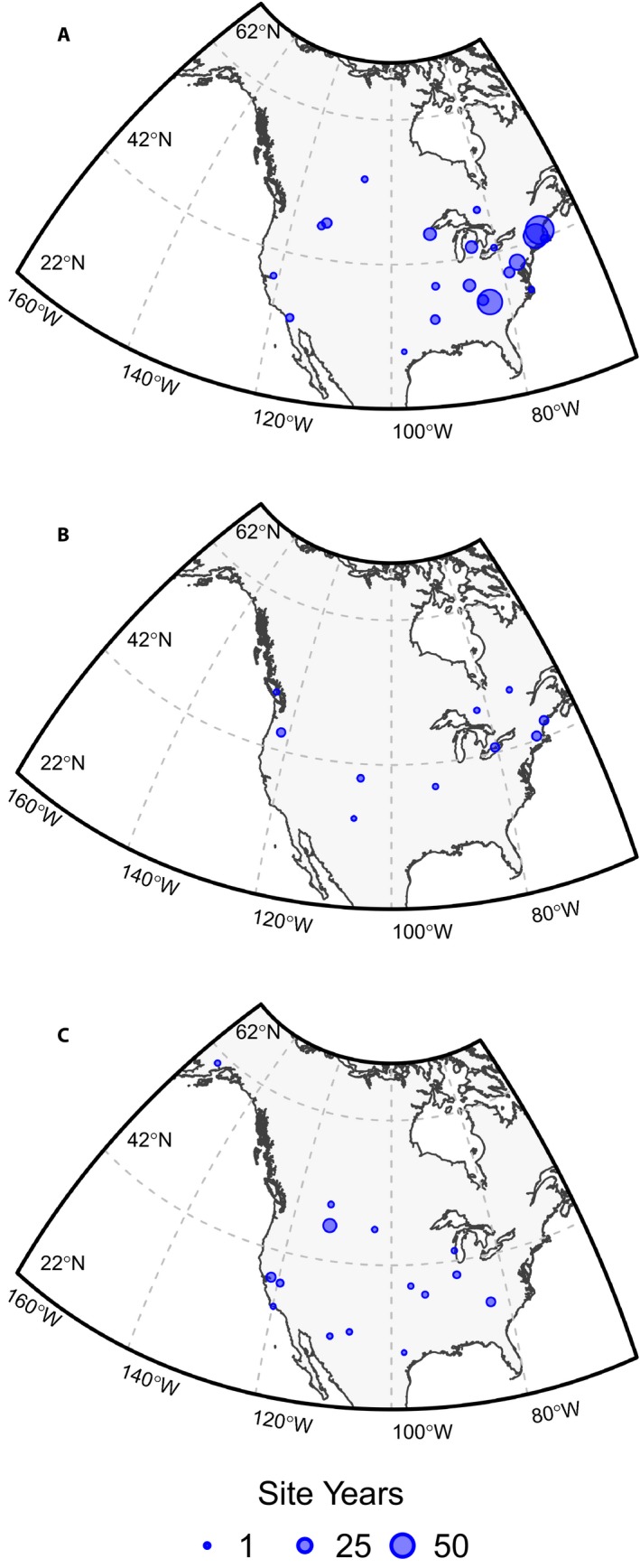
Location of PhenoCam sites used in this analysis, by vegetation type: deciduous broadleaf forest (DB) (A), evergreen needleleaf forest (EN) (B), and grassland (GR) (C). Data counts have been aggregated to a spatial resolution of 4°, and the size of each circle corresponds to the number of years of data within each cell.

Camera sites ranged across 30 degrees of latitude (29.9–60.6°N), with half of all sites between 38.4–44.5°N (Fig. 1). Sites ranged across more than 85 degrees of longitude (68–154°W), with elevations ranging from sea level to 3000 m above sea level. Sites spanned more than 20°C in mean annual temperature (−1.9–19.6°C) and a 10‐fold range in annual precipitation (255–2565 mm).

Deciduous broadleaf forests (DB) were best represented, with data from 65 camera sites, while for evergreen needleleaf forests (EN) and grasslands (GR) there were data from only about 20 sites per vegetation type (Table 1).

**Table 1:** Summary of PhenoCam sites included in this analysis, by vegetation type.^a^

Vegetation type	*N*	Transitions/site^b^		MAT (°C)	MAP (mm)	Latitude (°)	Longitude (°)	Elevation (m ASL)
		Rising	Falling					
DB	65	5.8	6.2	8.8 ± 3.9	1077 ± 304	41.3 ± 4.4	−82.2 ± 12.2	404 ± 384
EN	18	3.7	4.1	6.4 ± 3.3	1051 ± 451	43.6 ± 3.7	−90.1 ± 20.8	614 ± 793
GR	21	4.4	4.5	10.6 ± 5.7	671 ± 358	40.5 ± 7.1	−109.2 ± 16.2	748 ± 673

Note

ASL = above sea level; DB = deciduous broadleaf forest; EN = evergreen needleleaf forest; GR = grassland; MAP = mean annual precipitation; MAT = mean annual temperature; *N* = total number of sites for each vegetation type.

a Values are reported as mean ± 1 standard deviation.

b ”Rising” and “falling” indicate the mean number of transition dates, per site, for the “greenness rising” and “greenness falling” phenological transitions.

### Phenological patterns across sites

EN and GR sites tended to green‐up almost a month ahead of DB sites (Table 2). There was more than three times as much variation across GR sites in green‐up date, compared to across DB or EN sites. GR sites tended to green‐down a month and a half in advance of DB sites, and almost three months in advance of EN sites. There was three times as much variation across GR sites in green‐down dates, compared to across DB or EN sites.

**Table 2:** Mean phenological transition dates, and associated green season length, by vegetation type

Vegetation type			Green season length (days)^b^		
	Green‐up date^a^	Green‐down date^a^	Mean ± 1 SD	Minimum	Maximum
DB	115 ± 16	293 ± 21	178 ± 30	123	272
EN	87 ± 10	330 ± 21	244 ± 23	173	273
GR	95 ± 56	249 ± 66	152 ± 43	89	272

Note

DB = deciduous broadleaf forest; EN = evergreen needleleaf forest; GR = grassland; SD = standard deviation.

a ”Green‐up” and “Green‐down” dates are estimated according to the day of year on which 10% of the seasonal amplitude is passed during the “greenness rising” and “greenness falling” phenological transition periods. Values are mean day of year ± 1 standard deviation.

b Green season length (in days) is determined by the difference between green‐down date and green‐up date.

The duration of vegetation activity, measured as “green season length” (GSL = green‐down date – green‐up date), varied substantially both across and within vegetation types (Table 2). GR sites experienced the shortest duration of vegetation activity (mean ≈ 5 months) and EN sites the longest duration of vegetation activity (mean ≈ 8 months), with DB sites intermediate (mean ≈ 6 months). For each vegetation type, the longest observed GSL was almost exactly nine months.

**Figure 2:**
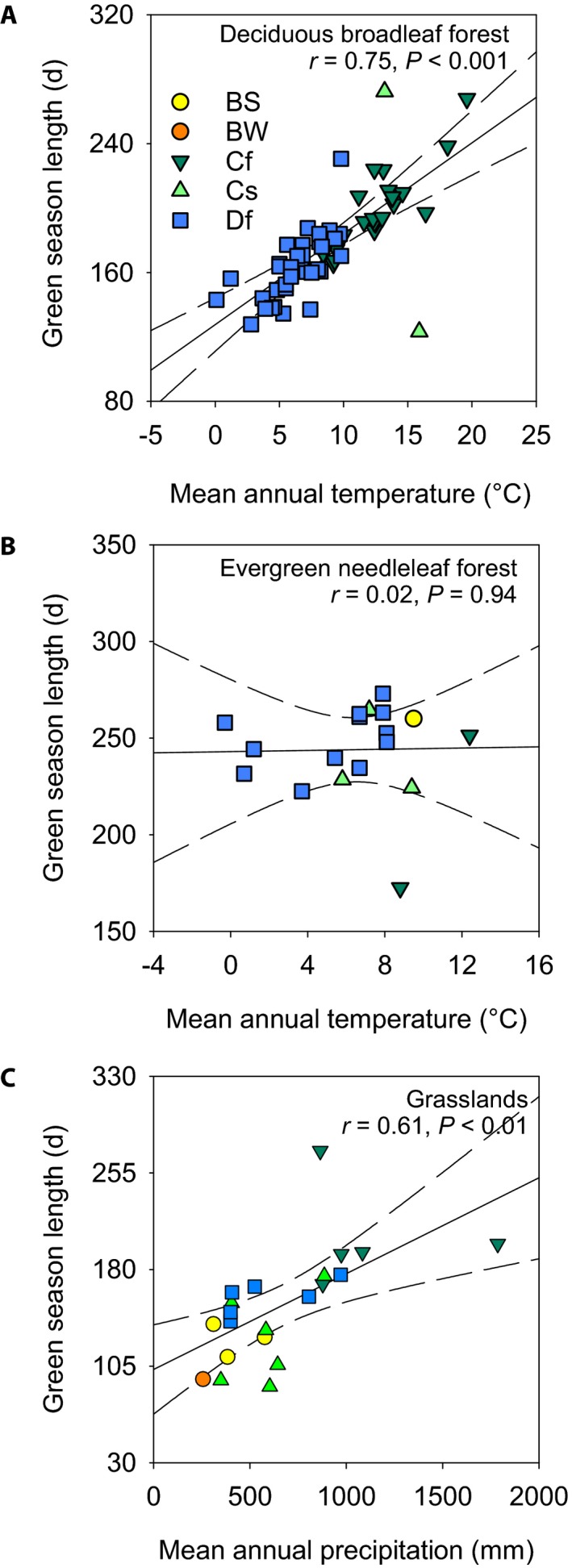
Spatial patterns of green season length in relation to climate variables, across different vegetation types: deciduous broadleaf forest (DB) (A), evergreen needleaf forest (EN) (B), and grassland (GR) (C). Symbols are colored according to the Köppen–Geiger climate classification: BS = arid steppe; BW = arid desert; Cf = temperate, without dry season; Cs = temperate, dry summer; Df = cold (continental), without dry season. Regression lines show best fit line and 99% confidence intervals.

Among different vegetation types, simple linear models differed in their ability to characterize the spatial patterns of phenological variability across sites (Tables 3, 4). The climate statistics model explained most of the spatial variation in DB green‐up date, and more than half of the spatial variation in DB GSL (Table 3). Most of this explanatory power was due to temperature, and for DB sites a 1°C increase in mean annual temperature was associated with a 4.0 ± 0.3 d advancement in the date of spring green‐up (partial *R* ^2^ = 0.74) and a 6.4 ± 0.7 d increase in GSL (partial *R* ^2^ = 0.56; Fig. 2A). The association between mean annual precipitation and DB phenology was also significant, as wetter sites tended to green‐up slightly later and have a marginally shorter GSL (opposite to what we found for DB sites in the interannual analysis, see below). By comparison, across EN sites, mean annual temperature and mean annual precipitation together explained very little of the observed phenological variability (total *R* ^2^ < 0.25 in all cases for EN), and mean annual temperature explained none of the spatial variability in EN GSL (Fig. 2B). For GR sites, spatial patterns were weakly explained by mean annual temperature, with warmer temperatures associated with both advanced green‐up (4.0 ± 1.9 d per 1°C; partial *R* ^2^ = 0.20) and advanced green‐down (5.4 ± 2.1 d per 1°C; partial *R* ^*2*^ = 0.19). For GR sites, neither green‐up date nor green‐down date was well explained by mean annual precipitation. And, because temperature shifted both green‐up and green‐down of GR sites in the same direction and by similar amounts, temperature was not strongly associated with spatial variation in GSL for this vegetation type. However, a 100‐mm increase in mean annual precipitation was associated with a 5.3 ± 1.2 d increase in GSL (partial *R* ^2^ = 0.46; Fig. 2C).

**Table 3:** Relationships between phenological events and climate statistics, by vegetation type.^a^,^b^

Vegetation type	Phenometric	*R* ^2^	RMSE	sMAT	*P* value	sMAP	*P* value
DB	Green‐up	0.81	6.8	−4.00 ± 0.25	<0.001	1.14 ± 0.25	<0.001
	Green‐down	0.20	16.9	2.41 ± 0.63	<0.001	−0.33 ± 0.63	0.61
	GSL	0.59	18.5	6.43 ± 0.69	<0.001	−1.49 ± 0.69	0.03
EN	Green‐up	0.04	8.9	0.18 ± 0.74	0.81	0.30 ± 0.53	0.59
	Green‐down	0.24	18.6	2.66 ± 1.53	0.10	−2.10 ± 1.11	0.08
	GSL	0.19	20.8	1.66 ± 1.72	0.35	−2.36 ± 1.25	0.08
GR	Green‐up	0.21	43.3	−4.03 ± 1.95	0.05	−0.74 ± 2.07	0.72
	Green‐down	0.34	46.0	−5.42 ± 2.11	0.02	4.50 ± 2.24	0.06
	GSL	0.52	25.0	−1.68 ± 1.16	0.17	5.29 ± 1.23	<0.001

*Note:*

DB = deciduous broadleaf forest; EN = evergreen needleleaf forest; GR = grassland; RMSE = root mean squared error of the linear model, in days; sMAP = sensitivity to mean annual precipitation (days per 100 mm); sMAT = phenological sensitivity to mean annual temperature (days per 1°C).

a “Green‐up” and “Green‐down” dates are estimated according to the day of year on which 10% of the seasonal amplitude is passed during the “greenness rising” and “greenness falling” phenological transition periods. Green season length (GSL, in days) is determined by the difference between green‐down date and green‐up date.

b Phenological sensitivities (mean ± 1 SE) are estimated across sites. Sites were weighted according to the number of years of observations used to calculate the site‐level mean.

**Table 4:** Relationships between phenological events and site location, by vegetation type.^a^,^b^

Vegetation type	Phenometric	*R* ^2^	RMSE	sLat	*P* value	sLon	*P* value	sElev	*P* value
DB	Green‐up	0.81	6.7	2.65 ± 0.20	<0.001	0.59 ± 0.07	<0.001	2.07 ± 0.20	<0.001
	Green‐down	0.23	16.7	−2.01 ± 0.49	<0.001	−0.11 ± 0.18	0.54	−1.01 ± 0.51	0.05
	GSL	0.61	18.3	−4.67 ± 0.53	<0.001	−0.71 ± 0.20	<0.001	−3.07 ± 0.56	<0.001
EN	Green‐up	0.04	9.3	−0.41 ± 0.87	0.64	−0.02 ± 0.13	0.87	0.06 ± 0.34	0.85
	Green‐down	0.63	13.4	−3.96 ± 1.25	<0.01	0.39 ± 0.19	0.06	−0.79 ± 0.49	0.13
	GSL	0.39	18.8	−2.96 ± 1.76	0.12	0.43 ± 0.26	0.12	−0.34 ± 0.70	0.63
GR	Green‐up	0.46	36.8	2.12 ± 1.51	0.18	0.87 ± 0.59	0.16	3.78 ± 1.29	<0.01
	Green‐down	0.60	37.9	3.30 ± 1.56	0.05	2.23 ± 0.61	<0.01	3.42 ± 1.32	0.02
	GSL	0.32	31.5	1.30 ± 1.30	0.33	1.38 ± 0.50	0.01	−0.08 ± 1.10	0.94

*Note:*

DB = deciduous broadleaf forest; EN = evergreen needleleaf forest; GR = grassland; RMSE = root mean squared error of the linear model, in days; sElev = sensitivity to increase in elevation (days per 100 m); sLat = sensitivity to longitude (days per 1° shift); sLon = sensitivity to longitude (days per 1° shift).

a “Green‐up” and “Green‐down” dates are estimated according to the day of year on which 10% of the seasonal amplitude is passed during the “greenness rising” and “greenness falling” phenological transition periods. Green season length (GSL, in days) is determined by the difference between green‐down date and green‐up date.

b Phenological sensitivities (mean ± 1 SE) are estimated across sites. Sites were weighted according to the number of years of observations used to calculate the site‐level mean.

**Figure 3:**
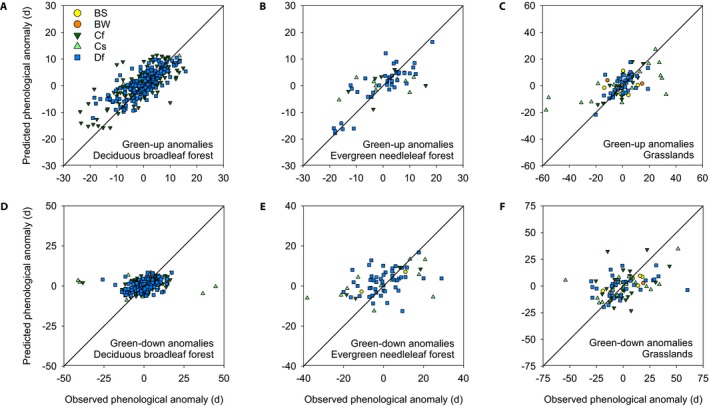
Correlation between observed and modeled (with stepwise linear regression) phenological anomalies (green‐up date anomalies [A–C] and green‐down date anomalies [D–F]), across different vegetation types: deciduous broadleaf forest (DB) (A, D), evergreen needleaf forest (EN) (B, E), and grassland (GR) (C, F). Model structure and regression statistics are reported in Table 7. (Note that observations were weighted according to the squared reciprocal of the estimated transition date uncertainty, but for clarity these uncertainties are not shown.) Symbols are colored according to the Köppen‐Geiger climate classification: BS = arid steppe; BW = arid desert; Cf = temperate, without dry season; Cs = temperate, dry summer; Df = cold (continental), without dry season. The diagonal line is the 1 : 1 line.

For DB sites, site location had substantial explanatory power for green‐up and, to a lesser degree, green‐down and GSL (Table 4). Although these patterns were dominated by the latitude effect (e.g., 2.6 ± 0.2 d delay of green‐up per °N, partial *R* ^2^ = 0.43; 2.0 ± 0.5 d advancement of green‐down per °N, partial *R* ^2^ = 0.18), longitude and elevation effects were also generally significant. Because we express longitude in decimal degrees, longitude is increasing (less negative) from west to east across North America. Thus, increasing longitude was associated with a 0.6 ± 0.1 d per °E delay in green‐up. Increasing elevation delayed green‐up (2.1 ± 0.2 d per 100 m) and advanced green‐down (1.0 ± 0.5 d per 100 m). The DB GSL was thus shorter at higher‐latitude, more easterly, and higher‐elevation sites. By comparison, for EN sites, latitude was only significantly associated with green‐down date (4.0 ± 1.2 d advancement of green‐down per °N) and not green‐up date or GSL; longitude and elevation were not significantly associated with any phenometric (Table 4). For the GR sites, results were more mixed, but the only phenometric for which location explained more than half of the observed across‐site variation in phenology was green‐down, which was delayed with increasing latitude north, delayed with increasing longitude west to east, and delayed with increasing elevation; note that the direction of these effects is in each case opposite to that for DB green‐down.

Our analysis of observed (*Syringa*) and modeled (SI‐x) phenological transitions—first leaf and first bloom—shows generally strong and surprisingly consistent relationships with site latitude, longitude, and elevation (Table 5), although a notable exception is that for *S. vulgaris* the longitude effect on first bloom was not significant (*P* = 0.07). When we restricted the wall‐to‐wall SI‐x analysis (Table 5) to those grid cells in which PhenoCams of specific vegetation types were also located (Table 6), the estimated site location model parameters shifted somewhat, but they remained more consistent with the wall‐to‐wall analysis than the PhenoCam‐based analysis (Tables 3, 4).

**Table 5:** Site location model applied to observed (*Syringa*) and modeled (SI‐x) phenological transitions.^a^^,^ b

Data source	Phenometric	*N*	*R* ^2^	sLat	sLong	sElev
*Syringa* ×*chinensis* (cloned)	First leaf	195	0.88	4.72 ± 0.14	0.64 ± 0.05	2.45 ± 0.22
	First bloom	194	0.92	4.43 ± 0.11	0.61 ± 0.04	1.70 ± 0.18
*Syringa vulgaris*	First leaf	725	0.81	3.45 ± 0.08	0.56 ± 0.07	2.68 ± 0.07
	First bloom	931	0.89	3.73 ± 0.05	−0.08 ± 0.04	2.80 ± 0.05
Spring Index (SI‐x) models	First leaf		0.94	4.88 ± 0.01	0.99 ± 0.01	2.53 ± 0.01
	First bloom		0.95	5.02 ± 0.01	0.87 ± 0.01	2.89 ± 0.01

a Phenological sensitivities (mean ± 1 SE) are estimated across sites: sLat, sLon = sensitivity to latitude and longitude (days per 1° shift); sElev = sensitivity to increase in elevation (days per 100 m).

b For observational data, *N* is the number of observational sites; for each site, multiple years of data were averaged to yield the site mean, which was used for analysis. For *Syringa* data, sites were weighted according to the number of years of observations used to calculate the site‐level mean.

**Table 6:** Site location model applied to Spring Index–modeled phenological transitions for lilac (*Syringa*) at PhenoCam site locations. In model A, site means were calculated across all years, 2000–2015; in model B, site means were calculated using only those years for which PhenoCam observations were available, with sites then weighted by the number of years of observations.^a^

PhenoCam vegetation type	SI‐x Phenometric	*R* ^2^	sLat	sLong	sElev
Site location model A					
DB	First leaf	0.96	5.37 ± 0.17	0.75 ± 0.06	1.85 ± 0.20
	First bloom	0.97	5.02 ± 0.14	0.73 ± 0.05	2.28 ± 0.17
EN	First leaf	0.97	4.96 ± 0.35	0.75 ± 0.05	2.34 ± 0.13
	First bloom	0.98	5.44 ± 0.29	0.49 ± 0.04	2.53 ± 0.12
GR	First leaf	0.97	5.24 ± 0.30	1.13 ± 0.11	1.75 ± 0.23
	First bloom	0.95	5.27 ± 0.42	1.09 ± 0.16	2.40 ± 0.32
Site location model B					
DB	First leaf	0.93	5.00 ± 0.16	0.78 ± 0.06	1.65 ± 0.19
	First bloom	0.94	4.93 ± 0.14	0.67 ± 0.05	2.06 ± 0.16
EN	First leaf	0.75	3.78 ± 1.19	0.68 ± 0.14	1.96 ± 0.35
	First bloom	0.86	4.62 ± 0.80	0.46 ± 0.09	2.28 ± 0.24
GR	First leaf	0.97	5.88 ± 0.32	1.20 ± 0.11	1.49 ± 0.25
	First bloom	0.94	5.76 ± 0.47	1.01 ± 0.17	2.36 ± 0.37

a Phenological sensitivities (mean ± 1 SE) are estimated across sites: sElev = sensitivity to increase in elevation (days per 100 m); sLat, sLon = sensitivity to latitude and longitude (days per 1° shift).

### Interannual variation in phenology

The ability of our stepwise regression model to reproduce observed phenological anomalies varied among vegetation types and between green‐up and green‐down (Fig. 3, Table 7). For example, DB green‐up was well predicted by the model (*R* ^2^ = 0.66), but DB green‐down was not (*R* ^2^ = 0.24); similarly mixed results were obtained for EN (green‐up *R* ^2^ = 0.78, green‐down *R* ^2^ = 0.43). For GR, green‐up and green‐down were both predicted with only moderate success (*R* ^2^ = 0.45 and *R* ^2^ = 0.39, respectively). The relative importance of temperature vs. precipitation also varied according to vegetation type and phenophase, but in all cases warmer temperatures consistently advanced green‐up and delayed green‐down. Notably, across all three vegetation types, outliers (Fig. 3) tended to be associated with temperate, dry summer (Köppen–Geiger Cs classification)—i.e., Mediterranean—sites. Although we included precipitation metrics in our stepwise modeling, a more mechanistic approach may be required in these seasonally water‐limited systems.

**Table 7:** Stepwise regression analysis of weather effects on phenological anomalies, by vegetation type and phenological transition

Vegetation type	Phenometric^a^	*k*	EDF	RMSE	*R* ^2^	Regression coefficients ± 1 SE^b^											
						$sT_{+11}^{+30}$	$sT_{-10}^{+10}$	$sT_{-30}^{-11}$	$sT_{-50}^{-31}$	$sT_{-70}^{-51}$	$sT_{-90}^{-71}$	$sP_{+11}^{+30}$	$sP_{-10}^{+10}$	$sP_{-30}^{-11}$	$sP_{-50}^{-31}$	$sP_{-70}^{-51}$	$s-P_{-90}^{-71}$
DB	Green‐up	6	357	3.8	0.66		−2.3 ± 0.1 (*P* < 0.001)	−1.1 ± 0.1 (*P* < 0.001)	−0.6 ± 0.1 (*P* < 0.001)					−1.2 ± 0.7 (*P* = 0.09)	−1.3 ± 0.6 (*P* = 0.02)		
	Green‐down	7	378	5.6	0.24		0.7 ± 0.2 (*P* < 0.01)	1.4 ± 0.2 (*P* < 0.001)	1.1 ± 0.2 (*P* < 0.001)		−0.9 ± 0.2 (*P* < 0.001)		1.9 ± 0.8 (*P* = 0.01)				−2.0 ± 0.9 (*P* = 0.02)
EN	Green‐up	4	51	4.5	0.78	−1.6 ± 0.6 (*P* < 0.01)	−1.9 ± 0.4 (*P* < 0.001)			−0.8 ± 0.3 (*P* = 0.02)							
	Green‐down	6	61	8.6	0.43		2.2 ± 0.5 (*P* < 0.001)		1.8 ± 0.8 (*P* = 0.03)				8.5 ± 2.8 (*P* < 0.01)		13.1 ± 3.3 (*P* < 0.001)		7.8 ± 3.9 (*P* = 0.05)
GR	Green‐up	7	81	10.4	0.45		−1.9 ± 0.8 (*P* = 0.01)	−1.5 ± 0.5 (*P* < 0.01)				6.7 ± 3.4 (*P* = 0.05)	9.0 ± 3.8 (*P* = 0.02)		−14.5 ± 2.9 (*P* < 0.001)		−7.5 ± 2.3 (*P* < 0.01)
	Green‐down	5	88	12.0	0.39	2.9 ± 0.7 (*P* < 0.001)							19.4 ± 5.5 (*P* < 0.001)		10.4 ± 3.3 (*P* < 0.01)	17.0 ± 3.8 (*P* < 0.001)	

Note

DB = deciduous broadleaf forest; EDF = error degrees of freedom; EN = evergreen needleleaf forest; GR = grassland; *k* = number of fit parameters (regression coefficients plus 1); RMSE = root mean squared error; *R* ^2^ = coefficient of multiple determination.

a Phenological transition dates—”green‐up” and “green‐down”—were estimated according to the day of year on which 10% of the seasonal amplitude is passed during the “greenness rising” and “greenness falling” phases.

b $sT_{x}^{x+19}$ is the phenological sensitivity to mean air temperature (in days per 1°C), calculated across a moving window (width = 20 d) offset by *x* days relative to the mean transition date. Negative values of *x* correspond to dates *before* the mean transition date, while positive values of *x* correspond to dates *after* the mean transition date. Similarly, $sP_{x}^{x+19}$ is the phenological sensitivity to total precipitation (in days per 100 mm). Regression coefficients are presented as estimated sensitivity ± 1 standard error, with *P* values in parentheses. Data points were weighted by the squared reciprocal of the estimated transition date uncertainty.

For DB and EN, temperature anomalies around the mean transition date were the most important predictors of phenological anomalies, while precipitation effects were relatively minor. For example, DB green‐up was strongly advanced by warmer concurrent ($sT_{-10}^{+10}$: −2.3 ± 0.8 d per 1°C, partial *R* ^2^ = 0.39) and antecedent ($sT_{-30}^{-11}$: −1.1 ± 0.1 d per 1°C, partial *R* ^2^ = 0.20; $sT_{-50}^{-31}$: −0.6 ± 0.1 d per 1°C, partial *R* ^2^ = 0.06) temperatures. Notably, the temperature sensitivity declined steadily the further in advance of mean green‐up date, to the point where temperatures more than 50 days in advance of mean green‐up were not significant factors in the regression. Intriguingly, summing these sensitivities to quantify the total effect of a sustained temperature anomaly yields an estimate of four days advancement per 1°C increase, which is identical to the spatial sensitivity of DB green‐up to mean annual temperature (Table 3). Although antecedent precipitation effects were found to be significant ($sP_{-30}^{-11}$, $sP_{-50}^{-31}$) for DB green‐up, they explained only a minuscule portion of the variation (both partial *R* ^2^ < 0.01). Likewise, antecedent temperatures delayed DB green‐down (e.g., $sT_{-30}^{-11}$: 1.4 ± 0.2 d per 1°C, partial *R* ^2^ = 0.14), although with lower explanatory power than in spring. Again, summing the estimated sensitivities to quantify the total effect of a sustained temperature anomaly yields an estimate of 2.3 days delay per 1°C increase, which is remarkably close to the spatial sensitivity of DB green‐down to mean annual temperature (2.7 ± 1.5 d delay per 1°C increase; Table 3). Although concurrent precipitation also delayed DB green‐down, the estimated sensitivity was very low, and the explanatory power almost negligible ($sP_{-10}^{+10}$: 1.9 ± 0.8 d per 100 mm, partial *R* ^2^ = 0.01). Thus, temperature, rather than precipitation, appears to be the dominant driver of interannual phenological variation at DB sites.

For EN, green‐up was also strongly advanced by warmer concurrent temperatures ($sT_{-10}^{+10}$: −1.9 ± 0.4 d per 1°C, partial *R* ^2^ = 0.71) but not precipitation, while green‐down was delayed both by warmer concurrent temperatures ($sT_{-10}^{+10}$: −2.2 ± 0.5 d per 1°C, partial *R* ^2^ = 0.19) and by increases in concurrent and antecedent precipitation ($sP_{-10}^{+10}$, $sP_{-50}^{-30}$, and $sP_{-90}^{-70}$ all positive; aggregate partial *R* ^2^ = 0.20).

For GR, the phenological sensitivities to weather were somewhat different compared to DB and EN, in that strong effects of both temperature and precipitation were consistently identified. For example, while warmer temperatures were again found to advance GR green‐up ($sT_{-10}^{+10}$: −1.9 ± 0.8 d per 1°C, partial *R* ^2^ = 0.12; $sT_{-30}^{-11}$: −1.5 ± 0.5 d per 1°C, partial *R* ^2^ = 0.06), the strongest effect was that of precipitation more than a month previous ($sP_{-50}^{-31}$: −14.5 ± 2.9 d per 100 mm, partial *R* ^2^ = 0.17), which also advanced green‐up. Likewise, while warmer temperatures were found to delay GR green‐down ($sT_{+11}^{+30}$: 2.9 ± 0.7 d per 1°C, partial *R* ^2^ = 0.10), antecedent precipitation—in this case, two months in the past ($sP_{-70}^{-51}$: 17.0 ± 3.8 d per 100 mm, partial *R* ^2^ = 0.13)—had the strongest effect, with increases in precipitation during this period associated with delayed GR green‐down. Increases in concurrent precipitation ($sP_{-10}^{+10}$: 19.4 ± 5.5 d per 100 mm, partial *R* ^2^ = 0.10) also delayed green‐down at a similar rate.

## DISCUSSION

### Assessment of the Bioclimatic Law

Our results show mixed support for the Bioclimatic Law. First, we found that not all phenophases and vegetation types followed the Law to the same degree. Although DB green‐up was well explained by latitude, longitude, and elevation, green‐down was less well explained by these factors (Table 4). EN and GR ecosystems did not adhere to the spirit of the Law, at least across the sites included in our analysis, as typically no more than one of latitude, longitude, or elevation was found to be statistically significant in the site location model (GR green‐down was an exception, but in this instance the regression coefficients were all opposite in sign compared to DB green‐down), and *R* ^2^ values rarely exceeded 0.5.

Second, our analysis of interannual patterns clearly shows that temperature and precipitation anomalies at particular times of the year can have very specific impacts on the observed phenological transitions. Thus, in a given year, when weather anomalies can be expected to be coherent at regional but not continental scales, departures from the Law over broad geographic gradients are also to be expected. Furthermore, while interannual variation in DB phenology was sensitive to temperature anomalies in a manner consistent with the spatial sensitivity of DB phenology to mean annual temperature—suggesting that spatial patterns might predict temporal patterns—the same was not true for EN and GR phenology.

How do our estimated site location model parameters for green‐up compare with those presented by Hopkins, or Hopp's (1976) later work on lilac cultivars? Whereas Hopkins reported delays of four days per °N and Hopp estimated 4.6 days per °N, we found the effect of latitude on DB green‐up to be substantially less steep (2.7 ± 0.2 days delay per °N). Hopkins reported delays of 0.8 days per °E, which was similar to our estimate (0.6 ± 0.1 days per °E), although not as steep as estimated by Hopp (1.3 days delay per °E). Finally, Hopkins reported delays of 3.3 days per 100 m increase in elevation, and Hopp estimated 2.6 days per 100 m, both of which are steeper than our estimate (2.1 ± 0.2 days delay per 100 m). Therefore, across the DB sites in our analysis, the estimated sensitivities of green‐up date to latitude, longitude, and elevation were substantially less steep than reported by Hopkins or Hopp, and together our estimated model parameters are significantly different from those presented by Hopkins (*F*_3,61_ = 23.4; *P* < 0.001).

Hopp's (1976) analysis suggests that the phenology of cloned lilac plants is closely aligned with gradients of latitude, longitude, and elevation, presumably because the environmental drivers of lilac phenology are also aligned with similar gradients. In our analysis, this also appeared to be the case for DB phenology. Our analysis of an expanded lilac data set (Table 5) was also generally consistent with Hopp's earlier analysis, although we saw some differences between *S. vulgaris* and *S*. ×*chinensis*. These differences may reflect the genetic homogeneity of *S*. ×*chinensis* vs. the heterogeneity of *S. vulgaris*, or they may be driven by differing climatic gradients in the western vs. eastern United States. For the wall‐to‐wall SI‐x model predictions (Table 5), latitudinal and elevational sensitivities were quite comparable to those estimated for *S*. ×*chinensis*, but the longitudinal effect was substantially stronger, perhaps because of the larger east–west gradient across the United States as a whole.

**Figure 4:**
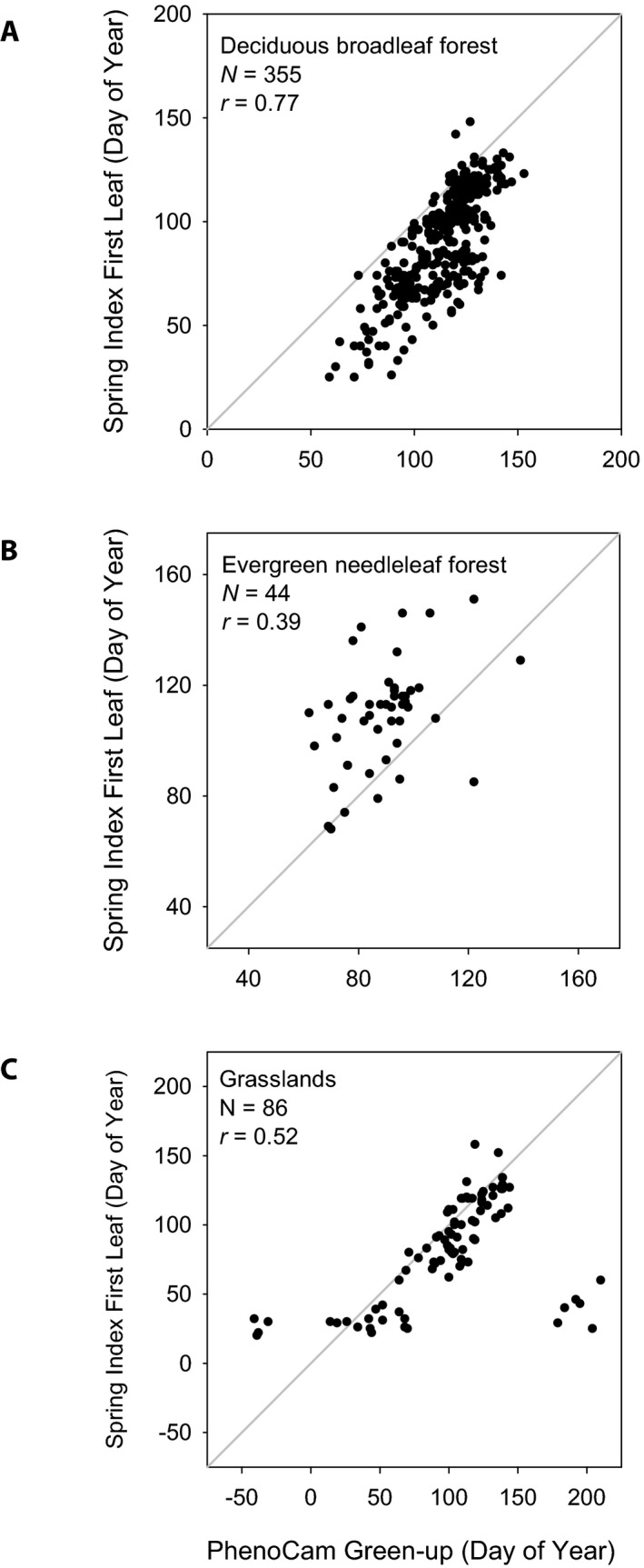
Comparison of “green‐up” date derived from PhenoCam imagery vs. Spring Index modeled “first leaf” date for deciduous broadleaf forest (A), evergreen needleleaf forest (B), and grasslands (C). For grasslands, the obvious outliers are generally water‐limited sites in the American Southwest. Excluding these, the correlation improves to *r* = 0.85.

A question that emerges from this analysis is why the EN and GR results are so different from those for DB and lilac. Is it because of the geographic and enviro‐climatic distribution of the specific sites used (i.e., sampling bias)? Or is it because the phenology of EN and GR vegetation responds to enviro‐climatic factors in a fundamentally different way (i.e., biology)? We conducted our analysis of SI‐x model predictions to assess whether the lack of strong spatial patterns for green‐up at EN and GR (Table 4) sites can be attributed to sampling bias, either in terms of the sites (Table 6) or years (Table 6) for which PhenoCam data are available. We found that, given the PhenoCam site locations, for each PhenoCam vegetation type the SI‐x model predictions correlate strongly with longitude, latitude, and elevation in a manner that is similar to the wall‐to‐wall SI‐x runs (Table 5). Thus, it appears that both spatial and temporal sampling would be sufficient to capture the covariation of phenology with latitude, longitude, and elevation at EN and GR sites, if the vegetation at those sites responded to environment in the same way that lilac does. The fact that such patterns are not seen in the PhenoCam‐derived green‐up data for EN and GR sites suggests that biology is the primary reason that EN and GR sites do not appear to follow the Bioclimatic Law. And thus, perhaps not surprisingly, across the sites we sampled, we see that DB green‐up dates are strongly correlated with SI‐x predicted “first leaf,” but this is not the case for EN or GR sites (Fig. 4).

For EN and GR sites, the poor performance of both the climate statistics model and the site location model may result from a variety of factors. It may be that while temperature is an important control on EN seasonality (Bowling et al., 2018; Richardson et al., 2018b), mean annual temperature is not the relevant metric. For GR sites, while temperature follows broad geographic gradients that correlate linearly with latitude, longitude, and elevation, precipitation—a key driver—does not follow the same patterns. Additionally, the environmental controls in different ecosystem types may vary (Seddon et al., 2016), e.g., water‐ vs. temperature‐limited grasslands. Alternatively, phenological variability may not correlate with either climatic gradients or geographic gradients because of species‐ and population‐specific phenological responses that run counter to (or simply do not align with) these gradients (Liang, 2016).

### Implications for modeling

The exploratory data analysis conducted here using stepwise regression can be used to generate hypotheses that can then be tested in new model formulations and evaluated against independent data. For example, our analysis of interannual variation in DB green‐up dates shows that the sensitivity to air temperature anomalies within 10 days of the mean budburst date is four‐fold higher than the sensitivity to air temperature anomalies a month in advance of the mean budburst date; we are not aware of this result having been previously reported. As noted by Friedl et al. (2014), this shows that the *timing* of thermal forcing is perhaps as important a control on budburst phenology as the *amount* of thermal forcing. Mechanistic models of deciduous tree budburst dates could most likely be substantially improved by accounting for this variation in temperature sensitivity.

Additionally, this analysis points to the need to develop improved process‐based phenological models, which account for more than just mean temperature by tracking state variables as a means of temporally integrating environmental signals and accounting for system memory (Hufkens et al., 2016). For example, in GR systems, balancing precipitation inputs against evapotranspiration and tracking metrics of site water balance (e.g., soil water content and/or climatic water deficit) are likely key. Beyond “thermal time” models that account for the states of chilling and forcing (Chuine, 2000), such model structures are not widely used to predict phenology because the data required for model parameterization have been difficult to obtain at a broad spatial scale. The method developed by Melaas et al. (2016)—whereby models are tuned to site‐level data and then validated at regional‐to‐biome scale using remote sensing—should prove useful. The recent analysis by Richardson et al. (2018c) demonstrates that for most ecosystem types camera‐ and satellite‐derived phenometrics are in close agreement, giving confidence in the use of remotely sensed data for validation. Phenological models could also be validated against transition dates estimated from herbarium samples collected years or decades earlier (Brenskelle et al., 2019; Pearson, 2019), enabling robust validation of hindcasting skill. Public release and documentation of software packages to facilitate phenological modeling (Hufkens et al., 2018; Park et al., 2019) should further contribute to advancing understanding of the controls on vegetation phenology.

## CONCLUSIONS

This analysis has shown the potential for using data from phenology cameras, which observe seasonal transitions in vegetation activity in an objective and consistent manner across diverse ecosystem and vegetation types (Brown et al., 2016), to inform empirical investigation of the enviro‐climatic controls on vegetation phenology. For example, for deciduous broadleaf forests, our analysis shows that spring green‐up correlates well with mean annual temperature and varies predictably along broad geographic gradients (consistent with the Bioclimatic Law), although interannual variation in phenology at individual sites is related to temperature anomalies during very specific windows of time. Ongoing expansion of the PhenoCam network will contribute to better representation of the diversity of ecosystems across the North American continent and the refinement of these results. Data from similar efforts focused on Asia (Nasahara and Nagai, 2015), Europe (Wingate et al., 2015), and Australia (Moore et al., 2016) can be used to conduct complementary analyses of the pheno‐climatology of other vegetation types worldwide.

## Supporting information


**APPENDIX S1.** Site characteristics of the PhenoCam sites used in the present study.

## Data Availability

PhenoCam imagery is publicly available through the project web page (http://phenocam.sr.unh.edu), and the PhenoCam Dataset v1.0 used in this study is publicly available through the ORNL DAAC (https://daac.ornl.gov/VEGETATION/guides/PhenoCam_V1.html; DOI: https://doi.org/10.3334/ORNLDAAC/1511).
