# Supplementary material for: Testing Hopkins’ Bioclimatic Law with PhenoCam data
Source: Appl Plant Sci. 2019 Mar 18;7(3):e01228. doi: 10.1002/aps3.1228 (PMC6426166; doi:10.1002/aps3.1228)
Supplement: Supplementary file 1 — APPENDIX S1. Site characteristics of the PhenoCam sites used in the present study. [file APS3-7-e01228-s001.docx]

**APPENDIX S1.** Site characteristics of the PhenoCam sites used in the present study. “Start date” is the date of the first image in the image archive. Imagery for all sites was processed through the end of 2015, unless the site was decommissioned prior to that date (“End date”). MAT is mean annual temperature, in °C; MAP is mean annual precipitation, in mm. Climate data are from WorldClim. Primary and secondary vegetation types are as follows: AG = agriculture; DB = deciduous broadleaf; DN = deciduous needleleaf; EB = evergreen broadleaf; EN = evergreen needleleaf; GR = grassland; MX = mixed vegetation (generally EN/DN, DB/EN, or DB/EB); SH = shrubs; TN = tundra (includes sedges, lichens, mosses, etc.); WL = wetland. IGBP landcover is according to MODIS land cover classification, following the International Geosphere-Biosphere Programme scheme (see Table 1).

| **Camera name** | **Full site name** | **Latitude (°)** | **Longitude (°)** | **Elevation (m ASL)** | **Start date** | **End**  **date** | **MAT (°C)** | **MAP (mm)** | **Primary**  **Veg.** | **Secondary Veg.** | **IGBP Landcover** | **Acknowledgments** |
| --- | --- | --- | --- | --- | --- | --- | --- | --- | --- | --- | --- | --- |
| acadia | Acadia National Park, McFarland Hill, near Bar Harbor, Maine | 44.3769 | −68.2608 | 158 | 2007-03-15 | 2015-12-31 | 6.5 | 1303 | DB | EN | 5 | Camera images from Acadia National Park are provided courtesy of the National Park Service Air Resources Program. |
| ahwahnee | Ahwahnee Meadow, Yosemite National Park, California | 37.7467 | −119.5816 | 1199 | 2008-08-28 | 2015-12-31 | 11.8 | 886 | EN | GR | 8 | Camera images from Yosemite National Park are provided courtesy of the National Park Service Air Resources Program. |
| alligatorriver | Alligator River National Wildlife Refuge, North Carolina | 35.7879 | −75.9038 | 1 | 2012-05-03 | 2015-12-31 | 16.4 | 1312 | DB | WL | 5 | Research at the Alligator River flux site is supported by DOE NICCR (award 08-SC-NICCR-1072), DOE-TES (awards 11-DE-SC-0006700 and 7090112), USDA Forest Service (award 13-JV-11330110-081) and USDA-NIFA (award 2014-67003-22068). |
| arbutuslake | Arbutus Lake, Huntington Wildlife Forest, Newcomb, New York | 43.9821 | −74.2332 | 535 | 2008-06-12 | 2014-08-23 | 4.8 | 1051 | DB | EN | 4 | Research at the Huntington Wildlife Forest sites is supported by the New York State Energy Research and Development Authority and the State University of New York, College of Environmental Science and Forestry. |
| ashburnham | Ashburnham State Forest / Overlook Middle School, Ashburnham, Massachusetts | 42.6029 | −71.9260 | 292 | 2011-09-13 | 2015-12-31 | 7.1 | 1147 | DB | EN | 5 |  |
| bartlett | Bartlett Experimental Forest, Bartlett, New Hampshire | 44.0646 | −71.2881 | 268 | 2005-10-04 | 2015-12-31 | 5.5 | 1224 | DB | EN | 5 | Research at the Bartlett Experimental Forest tower is supported by the National Science Foundation (grant DEB-1114804) and the USDA Forest Service's Northern Research Station. |
| bartlettir | Bartlett Experimental Forest, Bartlett, New Hampshire | 44.0646 | −71.2881 | 268 | 2008-04-18 | 2015-12-31 | 5.5 | 1224 | DB | EN | 5 | Research at the Bartlett Experimental Forest tower is supported by the National Science Foundation (grant DEB-1114804) and the USDA Forest Service's Northern Research Station. |
| bitterootvalley | Bitterroot Valley, Stephensville, Montana | 46.5070 | −114.0910 | 1017 | 2002-04-18 | 2015-12-31 | 7.2 | 318 | DB |  | 13 | Camera images from the Bitterroot Valley are provided courtesy of the USDA Forest Service Air Resources Management Program. |
| bostoncommon | Boston Common, Boston, Massachusetts | 42.3559 | −71.0641 | 10 | 2010-05-06 | 2015-12-31 | 9.8 | 1127 | DB |  | 13 | Research at the Boston Common is supported by an Emerson College Faculty Advancement Funds Grant. |
| bostonu | Boston University, Charles River Esplanade, Boston, Massachusetts | 42.3504 | −71.1044 | 10 | 2012-09-14 | 2015-12-31 | 9.7 | 1121 | DB |  | 13 |  |
| boundarywaters | Boundary Waters Canoe Area Wilderness, Superior National Forest, Minnesota | 47.9467 | −91.4955 | 519 | 2005-12-16 | 2015-12-31 | 2.8 | 719 | DB | EN | 5 | Camera images from Superior National Forest are provided courtesy of the USDA Forest Service Air Resources Management Program. |
| bullshoals | Bull Shoals Field Station, Missouri State University, Drury-Mincy Conservation Area, M | 36.5628 | −93.0666 | 260 | 2013-11-20 | 2015-12-31 | 13.9 | 1084 | DB | GR | 4 |  |
| butte | Continental Divide, Butte, Montana | 45.9530 | −112.4796 | 1682 | 2008-04-01 | 2015-12-31 | 4.3 | 311 | GR | SH | 10 | Research at the Continental Divide PhenoCam Site in Butte, Montana, is supported by the National Science Foundation-EPSCoR (grant NSF-0701906), OpenDap, Inc., and Montana Tech of the University of Montana |
| canadaOA | BERMS Old Aspen Site, Prince Albert National Park, Saskatchewan, Canada | 53.6289 | −106.1978 | 601 | 2011-06-16 | 2015-12-31 | 0.1 | 445 | DB |  | 5 | Research at the Old Aspen Flux Tower site is supported through the Changing Cold Regions Network, with funding from the National Science and Engineering Research Council of Canada (NSERC), Greg Neufeld/Eagle Bay Resort |
| caryinstitute | Cary Institute of Ecosystem Studies, Millbrook, NY | 41.7839 | −73.7341 | 127 | 2008-04-15 | 2015-12-31 | 8.9 | 1084 | DB |  | 4 |  |
| cedarcreek | Cedar Creek Ecosystem Science Reserve (LTER), East Bethel, Minnesota | 45.4019 | −93.2042 | 276 | 2009-12-01 | 2010-08-10 | 6.3 | 750 | DB |  | 14 | Research at Cedar Creek Ecosystem Science Reserve is supported by the National Science Foundation LTER program and the Minnesota Agriculture Experiment Station. |
| chibougamau | Eastern Old Black Spruce Site, Chibougamau, Quebec, Canada | 49.6924 | −74.3420 | 380 | 2008-03-19 | 2011-11-22 | -0.3 | 970 | EN |  | 1 |  |
| coaloilpoint | Coal Oil Point Natural Reserve, Santa Barbara, California | 34.4137 | −119.8802 | 6 | 2008-05-11 | 2012-12-05 | 15.4 | 405 | GR | SH | 7 |  |
| columbiamissouri | University of Missouri, Ashland Wildlife Research Area, Missouri | 38.7441 | −92.1997 | 232 | 2006-11-18 | 2009-01-23 | 12.4 | 974 | DB | EN | 4 |  |
| coweeta | Coweeta Hydrologic Laboratory, USDA Forest Service, Southern Research Station, Otto, N | 35.0596 | −83.4280 | 680 | 2011-04-08 | 2015-12-31 | 12.5 | 1722 | DB |  | 5 | Research at the Coweeta flux tower is funded through the USDA Forest Service, Southern Research Station; USDA Agriculture and Food Research Initiative Foundational Program, award number 2012-67019-19484; EPA agreement number 13-IA-11330140-044; and the National Science Foundation, Long-Term Ecological Research (LTER) program, award #DEB-0823293. |
| dollysods | Canaan Valley / Dolly Sods Wilderness, Monongahala National Forest, West Virginia | 39.0995 | −79.4270 | 1133 | 2003-11-21 | 2015-12-31 | 7.6 | 1328 | DB |  | 4 | Camera images from the Dolly Sods Wilderness, Mononghahela National Forest are provided courtesy of the USDA Forest Service Air Resources Management Program. |
| downerwoods | UW-Milwaukee Field Station, Downer Woods Natural Area, Milwaukee, Wisconsin | 43.0794 | −87.8808 | 213 | 2013-03-25 | 2015-12-31 | 8.2 | 812 | DB |  | 13 |  |
| drippingsprings | Cleveland National Forest, California | 33.3000 | −116.8000 | 400 | 2001-04-06 | 2009-05-26 | 13.2 | 599 | DB | SH | 8 | Camera images from Cleveland National Forest are provided courtesy of the USDA Forest Service Air Resources Management Program. |
| dukehw | Hardwood Stand, Duke Forest, North Carolina | 35.9736 | −79.1004 | 400 | 2013-05-31 | 2015-12-31 | 14.6 | 1166 | DB |  | 5 |  |
| freemangrass | Grassland Site, Texas State University, Freeman Ranch, San Marcos, Texas | 29.9300 | −98.0100 | 243 | 2012-03-13 | 2014-02-27 | 19.6 | 865 | GR | DB | 9 |  |
| freemanwood | Woodland Site, Texas State University, Freeman Ranch, San Marcos, Texas | 29.9400 | −97.9900 | 254 | 2012-06-27 | 2014-03-21 | 19.6 | 862 | DB | EN | 8 |  |
| gatesofthemountains | Gates of the Mountains Wilderness, Helena National Forest, Montana | 46.8262 | −111.7107 | 2387 | 2001-06-05 | 2015-12-31 | 1.1 | 577 | EN | GR | 1 | Camera images from Helena National Forest are provided courtesy of the USDA Forest Service Air Resources Management Program. |
| groundhog | Groundhog River, Ontario, Canada | 48.2174 | −82.1555 | 350 | 2008-10-21 | 2014-05-17 | 1.2 | 761 | EN | DB | 5 | Research at the Groundhog River site was supported by the Natural Sciences and Engineering Research Council (NSERC), the Canadian Foundation for Climate and Atmospheric Sciences (CFCAS), Great Lakes Forestry Centre (GLFC) of the Canadian Forest Service (CFS), |
| harvard | EMS Tower, Harvard Forest, Petersham, Massachusetts | 42.5378 | −72.1715 | 340 | 2008-04-04 | 2015-12-31 | 6.8 | 1139 | DB | EN | 5 | Research at Harvard Forest is partially supported through the National Science Foundation's LTER program (DEB-1237491), and Dept. of Energy Office of Science (BER) |
| harvardbarn | Barn Tower, Camera 1, Harvard Forest, Petersham, Massachusetts | 42.5353 | −72.1899 | 350 | 2011-07-27 | 2015-12-31 | 6.7 | 1151 | EN | DB | 5 | Research at Harvard Forest is partially supported through the National Science Foundation's LTER program (DEB-1237491). |
| harvardbarn2 | Barn Tower, Camera 2, Harvard Forest, Petersham, Massachusetts | 42.5353 | −72.1899 | 350 | 2011-08-03 | 2015-12-31 | 6.7 | 1151 | DB | EN | 5 | Research at Harvard Forest is partially supported through the National Science Foundation's LTER program (DEB-1237491). |
| harvardblo | Below-canopy camera, EMS Tower, Harvard Forest, Petersham, Massachusetts | 42.5378 | −72.1715 | 340 | 2009-04-01 | 2015-12-31 | 6.8 | 1139 | DB | EN | 5 | Research at Harvard Forest is partially supported through the National Science Foundation's LTER program (DEB-1237491). |
| harvardhemlock | Hemlock Tower, Harvard Forest, Petersham, Massachusetts | 42.5390 | −72.1800 | 355 | 2010-06-08 | 2015-12-31 | 6.7 | 1151 | EN | DB | 5 | Research at Harvard Forest is partially supported through the National Science Foundation's LTER program (DEB-1237491), and Dept. of Energy Office of Science (BER) |
| harvardlph | LPH Tower, Harvard Forest, Petersham, Massachusetts | 42.5420 | −72.1850 | 380 | 2010-06-15 | 2015-12-31 | 6.8 | 1139 | DB | EN | 5 | Research at Harvard Forest is partially supported through the National Science Foundation's LTER program (DEB-1237491). |
| howland1 | Main Tower (Mature stand), Howland Forest, Howland, Maine | 45.2041 | −68.7403 | 80 | 2007-01-01 | 2015-12-31 | 5.4 | 1056 | EN | DB | 5 | Research at Howland Forest is supported by the Office of Science (BER), US Department of Energy, and the USDA Forest Service's Northern Research Station. |
| howland2 | North Tower (Regrowing clearcut, ca. 1990), Howland Forest, Howland, Maine | 45.2128 | −68.7418 | 79 | 2008-03-30 | 2015-12-31 | 5.3 | 1058 | DB | EN | 5 | Research at Howland Forest is supported by the Office of Science (BER), US Department of Energy, and the USDA Forest Service's Northern Research Station. |
| hubbardbrook | Hubbard Brook Experimental Forest, USDA Forest Service Headquarters, North Woodstock, | 43.9438 | −71.7010 | 253 | 2008-04-16 | 2015-12-31 | 5.6 | 1060 | DB |  | 4 | Research at the Hubbard Brook Experimental Forest is partially supported by the National Science Foundation's LTER program (grant DEB-1114804) and the USDA Forest Service's Northern Research Station |
| hubbardbrooknfws | North Facing Watersheds, Hubbard Brook Experimental Forest, Thornton, New Hampshire | 42.9580 | −71.7762 | 930 | 2012-09-24 | 2015-12-31 | 7.4 | 1111 | DB |  | 5 | The Hubbard Brook Ecosystem Study is a collaborative effort at the Hubbard Brook Experimental Forest, which is operated and maintained by the USDA Forest Service, Northern Research Station, Newtown Square, PA. |
| hubbardbrooksfws | South Facing Watersheds, Hubbard Brook Experimental Forest, Thornton, New Hampshire | 43.9269 | -71.7407 | 650 | 2012-03-05 | 2015-12-31 | 4.6 | 1190 |  |  | 5 | The Hubbard Brook Ecosystem Study is a collaborative effort at the Hubbard Brook Experimental Forest, which is operated and maintained by the USDA Forest Service, Northern Research Station, Newtown Square, PA. |
| ibp | Jornada Experimental Range, New Mexico | 32.5890 | −106.8470 | 1325 | 2013-05-10 | 2015-12-31 | 14.8 | 257 | GR | SH | 7 | This research at the Jornada Experimental Range is funded by the USDA-Agriculture Research Service (ARS) via ARS Project Number 3050-11210-007-00D. Select camera locations are co-located with sites funded by National Science Foundation under Grant number DEB 1235828 as part of the Jornada Basin LTER program to New Mexico State University. |
| jasperridge | Jasper Ridge Biological Preserve, Woodside, California | 37.4020 | −22.2210 | 197 | 2012-02-29 | 2015-12-31 | 13.7 | 644 | GR |  | 8 | Supported by Jasper Ridge Biological Preserve of Stanford University and the Carnegie Institution Department of Global Ecology |
| joycekilmer | Joyce Kilmer Slickrock Wilderness, North Carolina | 35.2570 | −83.7950 | 1373 | 2006-06-06 | 2015-12-31 | 9.2 | 1905 | DB |  | 4 | Camera images from the Joyce Kilmer Slickrock Wilderness are provided courtesy of the USDA Forest Service Air Resources Management Program. |
| kansas | KU Field Station, University of Kansas, Kansas | 39.0561 | −95.1907 | 333 | 2012-03-17 | 2015-12-31 | 12.5 | 974 | GR |  | 14 | The US-KFS site is sponsored by the U.S. Department of Energy under a sub contract from DE-AC02-05CH11231. |
| kendall | Kendall Grassland, Arizona | 31.7365 | −109.9419 | 1529 | 2012-07-06 | 2015-12-31 | 15.9 | 384 | GR |  | 10 | Research at Walnut Gulch Experimental Watershed is funded by the USDA-ARS. The Kendall Ameriflux core site is also supported by the Dept. Of Energy Office of Science. |
| konza | Konza Prairie Biological Station, Kansas State University, Kansas | 39.0824 | −96.5603 | 443 | 2012-03-17 | 2015-12-31 | 11.8 | 878 | GR |  | 10 | The US-Kon site acknowledges support from the LTER program at the Konza Prairie Biological Station (DEB-0823341), and the U.S. Department of Energy under a sub contract from DE-AC02-05CH11231. |
| laurentides | Station de biologie des Laurentides, UniversityÂ of Montreal, St-Hippolyte,Â Quebec, C | 45.9881 | −74.0055 | 350 | 2011-09-16 | 2015-12-31 | 3.7 | 1066 | DB |  | 5 |  |
| lethbridge | Lethbridge Grassland Ecosystem Site, Lethbridge, Alberta | 49.7092 | −112.9403 | 950 | 2011-12-07 | 2015-12-31 | 5.1 | 399 | GR |  | 10 | Research at the Lethbridge Grassland Ecosystem site is supported by grants from the Natural Sciences and Engineering Research Council of Canada (RGPIN-2014-05882) to L.B. Flanagan. |
| mammothcave | Environmental Learning Center, Mammoth Cave National Park, Kentucky | 37.1858 | −86.1019 | 226 | 2002-01-01 | 2015-12-31 | 13.5 | 1312 | DB |  | 4 | Camera images from Mammoth Cave National Park are provided courtesy of the National Park Service Air Resources Program. |
| missouriozarks | University of Missouri, Ashland Wildlife Research Area, Missouri | 38.7441 | −92.2000 | 219 | 2012-03-05 | 2015-12-31 | 12.4 | 974 | DB |  | 4 | Research at the MOFLUX site is supported by the U.S. Department of Energy, Office of Science, Office of Biological and Environmental Research Program, Climate and Environmental Sciences Division. ORNL is managed by UT-Battelle, LLC, for the U.S. Department of Energy under contract DE-AC05-00OR22725. U.S. Department of Energy support for the University of Missouri (Grant DE-FG02-03ER63683) is gratefully acknowledged. |
| monture | Lolo National Forest, Ovando, Montana | 47.0202 | −113.1283 | 1255 | 2001-06-08 | 2015-12-31 | 5.0 | 407 | GR | DB | 12 | Camera images from Lolo National Forest are provided courtesy of the USDA Forest Service Air Resources Management Program. |
| morganmonroe | Morgan Monroe State Forest, Indiana | 39.3231 | -86.4131 | 275 | 2008-08-27 | 2015-12-31 | 11.2 | 1087 | DB |  | 4 | Research at the Morgan-Monroe Ameriflux site is supported by the US Departement of Energy, Office of Science, Office of Biological and Environmental Research throuth the Ameriflux Management Project administered by Lawrence Berkeley National Lab |
| nationalcapital | Park Police Headquarters, National Capital Parks, Washington DC | 38.8882 | −77.0695 | 28 | 2003-07-25 | 2015-12-31 | 13.1 | 1020 | DB |  | 13 | Camera images from the National Capital are provided courtesy of the National Park Service Air Resources Program. |
| niwot2 | Niwot Ridge Mountain Research Station, Roosevelt National Forest, Colorado | 40.0329 | −105.5470 | 3050 | 2009-07-23 | 2015-07-16 | 0.7 | 654 | EN |  | 1 | The US-NR1 AmeriFlux site is currently supported by the U.S. DOE, Office of Science through the AmeriFlux Management Project (AMP) at Lawrence Berkeley National Laboratory under Award Number 7094866. |
| northattleboroma | North Attleboro High School, North Attleboro, Massachusetts | 41.9837 | −71.3106 | 60 | 2012-02-21 | 2015-12-31 | 9.4 | 1202 | DB |  | 14 |  |
| oakridge1 | Chestnut Ridge, Oak Ridge, Tennessee | 35.9311 | −84.3323 | 371 | 2006-10-12 | 2015-12-31 | 13.8 | 1365 | DB |  | 4 | Research at Chestnut Ridge is funded by US Dept of Commerce, National Oceanic and Atmospheric Administration, Office of Atmospheric Research, Air Resources Lab, Atmospheric Turbulence and Diffusion Division as part of the Surface Energy Budget Network (SEBN) |
| oakridge2 | Chestnut Ridge, Oak Ridge, Tennessee | 35.9311 | −84.3323 | 371 | 2006-10-12 | 2015-12-31 | 13.8 | 1365 | DB |  | 4 | Research at Chestnut Ridge is funded by US Dept of Commerce, National Oceanic and Atmospheric Administration, Office of Atmospheric Research, Air Resources Lab, Atmospheric Turbulence and Diffusion Division as part of the Surface Energy Budget Network (SEBN) |
| oregonMP | Metolius intermediate pine/US-Me2, near Sisters, Oregon | 44.4523 | −121.5574 | 1253 | 2011-06-14 | 2015-12-31 | 5.8 | 893 | EN |  | 1 | Support for US-Me2 is provided from the Metolius Core Site Cluster by the DOE Office of Science Ameriflux Network Management Project |
| oregonYP | Metolius New Young Pine/US-Me6, near Sisters, Oregon | 44.3238 | −121.6060 | 977 | 2011-08-18 | 2015-12-31 | 7.2 | 570 | EN |  | 1 | Support for US-Me6 is provided from the Metolius Core Site Cluster by the DOE Office of Science Ameriflux Network Management Project |
| proctor | University of Vermont, Proctor Maple Research Center, Underhill, Vermont | 44.5250 | −72.8660 | 403 | 2008-09-11 | 2015-12-31 | 5.0 | 1081 | DB |  | 4 | Supported by the Agricultural Experiment Station of the University of Vermont |
| queens | Queen's University Biological Station, Lake Opinicon, Ontario, Canada | 44.5650 | −76.3240 | 126 | 2008-05-26 | 2015-12-31 | 6.4 | 887 | DB |  | 5 |  |
| readingma | Austin Prep School, Reading, Massachusetts | 42.5304 | −71.1272 | 100 | 2012-03-06 | 2015-12-31 | 9.3 | 1107 | DB |  | 13 |  |
| russellsage | Russell Sage State Wildlife Management Area, near Monroe, Louisiana | 32.4570 | −91.9743 | 20 | 2013-11-20 | 2015-12-31 | 18.1 | 1341 | DB |  | 5 |  |
| sanford | Sanford Natural Area, Michigan State University, East Lansing, Michigan | 42.7268 | −84.4645 | 268 | 2013-12-20 | 2015-12-31 | 8.1 | 781 | DB |  | 13 |  |
| shalehillsczo | Susquehanna Shale Hills Critical Zone Observatory (CZO), Pennsylvania | 40.6500 | −77.9000 | 310 | 2012-04-18 | 2015-12-31 | 9.8 | 980 | DB |  | 4 | Research at the Penn State Sone Valley Forest is supported by the National Science Foundation EAR 07-25019 (C. Duffy), and EAR 12-39285, EAR 13-31726 (S. Brantley) for the Susquehanna Shale Hills Critical Zone Observatory and the College of Agricultural Sciences, Department of Ecosystem Science and Management. |
| shenandoah | Pinnacles Viewpoint, Shenandoah National Park, Virginia | 38.6167 | −78.3500 | 1037 | 2009-09-14 | 2015-12-31 | 8.4 | 1222 | DB |  | 4 | Funding for the Shenandoah PhenoCam and related research has been provided by the U.S. Geological Survey Land Change Science Program (Shenandoah National Park Phenology Project) with logistical support from the National Park Service in collaboration with the University of Virginia Department of Environmental Sciences. |
| sherman | Twitchell Island, Antioch, California | 38.0366 | −121.7540 | -5 | 2014-01-01 | 2015-05-02 | 15.5 | 349 | GR |  | 12 |  |
| shiningrock | Shining Rock Wilderness, Blue Ridge Parkway National Park, North Carolina | 35.3902 | −82.7750 | 1500 | 2000-08-09 | 2015-12-31 | 9.3 | 1835 | DB |  | 4 | Camera images from the Shining Rock Wilderness are provided courtesy of the USDA Forest Service Air Resources Management Program. |
| silaslittle | Silas Little Experimental Forest, New Lisbon, New Jersey | 39.9137 | −74.5960 | 33 | 2011-03-15 | 2015-12-31 | 11.6 | 1128 | DB |  | 5 | Research at the Silas Little Experimental Forest is supported by the USDA Forest Service, Northern Research Station, NRS-06, Climate, Fire, and Carbon Cycle Sciences. |
| smokylook | Look Rock, Great Smoky National Park, Tennessee | 35.6325 | −83.9431 | 801 | 2000-02-11 | 2015-12-31 | 12.3 | 1487 | DB |  | 4 | Camera images from Great Smoky National Park are provided courtesy of the National Park Service Air Resources Program. |
| smokypurchase | Purchase Knob, Great Smoky National Park, Tennessee | 35.5900 | −83.0775 | 1550 | 2003-08-19 | 2015-12-31 | 8.7 | 1786 | DB | GR | 4 | Camera images from Great Smoky National Park are provided courtesy of the National Park Service Air Resources Program. |
| snakerivermn | Hay-Snake State Wildlife Management Area, near Woodland, Minnesota | 46.1206 | −93.2447 | 1181 | 2009-11-18 | 2015-12-31 | 4.4 | 742 | DB |  | 5 |  |
| snipelake | Snipe Lake, Lake Clark National Park and Preserve, Alaska | 60.6103 | −154.3199 | 706 | 2010-08-12 | 2015-06-10 | -1.9 | 525 | GR | TN | 7 |  |
| springfieldma | Academy Hill School, Springfield, Massachusetts | 42.1352 | −72.5860 | 56 | 2012-02-21 | 2015-12-31 | 9.3 | 1115 | DB |  | 13 |  |
| teddy | Painted Canyon Visitor Center, Teddy Roosevelt National Park, North Dakota | 46.8947 | −103.3775 | 853 | 2002-08-20 | 2015-12-31 | 5.7 | 399 | SH | GR | 10 | Camera images from Teddy Roosevelt National Park are provided courtesy of the National Park Service Air Resources Program. |
| thompsonfarm2N | University of New Hampshire, Thompson Farm Observatory, Durham, New Hampshire | 43.1086 | −70.9505 | 23 | 2009-01-11 | 2015-12-31 | 8.1 | 1108 | DB | EN | 5 | Research at the Thompson Farm Observatory is supported by NH EPSCoR with support from the National Science Foundation's Research Infrastructure Improvement Award (#EPS 1101245) and by the NH Agricultural Experiment Station/USDA NIFA (Hatch project #1006997). |
| tonzi | Tonzi Ranch, Amador County, California | 38.4309 | −120.9659 | 177 | 2011-10-26 | 2015-12-31 | 15.9 | 603 | DB | GR | 9 | Funding for AmeriFlux core site data was provided by the U.S. Department of Energy's Office of Science. |
| turkeypointdbf | Mature Deciduous Site, Turkey Point Carbon Cycle Research Project, Ontario, Canada | 42.6353 | −80.5576 | 211 | 2012-02-10 | 2015-12-31 | 8.0 | 968 | DB |  | 4 |  |
| turkeypointenf02 | 2002 White Pine, Turkey Point Carbon Cycle Research Project, Ontario, Canada | 42.6609 | −80.5595 | 194 | 2012-01-13 | 2015-12-31 | 8.1 | 963 | EN | AG | 14 |  |
| turkeypointenf39 | 1939 White Pine, Turkey Point Carbon Cycle Research Project, Ontario Canada | 42.7098 | −80.3574 | 232 | 2012-01-11 | 2015-12-31 | 7.9 | 973 | EN |  | 5 |  |
| turkeypointenf74 | 1974 White Pine, Turkey Point Carbon Cycle Research Project, Ontario Canada | 42.7068 | −80.3483 | 216 | 2012-02-02 | 2015-12-31 | 7.9 | 974 | EN |  | 5 |  |
| uiefprairie | Restored prairie at the University of Illinois Energy Farm, Urbana, IL USA | 40.0637 | −88.1973 | 224 | 2008-10-22 | 2015-12-31 | 10.9 | 971 | GR |  | 12 | The UIUC Energy Farm flux towers are supported by the Global Change and Photosynthesis Research Unit of the USDA Agricultural Research Service. |
| umichbiological | University of Michigan Biological Station, near Pellston, Michigan | 45.5598 | −84.7138 | 230 | 2008-08-21 | 2015-12-31 | 5.9 | 797 | DB | EN | 4 | Primary support for the University of Michigan AmeriFlux Core Site (US-UMB) provided by the Department of Energy Office of Science. Infrastructure support provided by the University of Michigan Biological Station. |
| umichbiological2 | FASET Tower, University of Michigan Biological Station, near Pellston, Michigan | 45.5625 | −84.6976 | 240 | 2008-11-24 | 2015-12-31 | 5.9 | 797 | DB | EN | 5 | Primary support for the University of Michigan AmeriFlux Core Site (US-UMd) provided by the Department of Energy Office of Science. Infrastructure support provided by the University of Michigan Biological Station. |
| upperbuffalo | Upper Buffalo Wilderness, Ozark National Forest, Arkansas | 35.8637 | −93.4932 | 777 | 2005-11-02 | 2015-12-31 | 13.0 | 1247 | DB |  | 4 | Camera images from Ozark National Forest are provided courtesy of the USDA Forest Service Air Resources Management Program. |
| usgsreston | USGS Headquarters, Reston, Virginia | 38.9471 | −77.3676 | 10 | 2012-03-09 | 2014-06-16 | 12.4 | 1019 | DB |  | 13 |  |
| usmpj | Heritage Land Conservancy, Pinyon-Juniper Site near Mountainair, NM | 34.4385 | −106.2544 | 2126 | 2013-09-20 | 2015-12-31 | 9.5 | 424 | SH | EN | 7 |  |
| uwmfieldsta | University of Wisconsin-Milwaukee Field Station, Saukville, Wisconsin | 43.3871 | −88.0229 | 265 | 2013-03-14 | 2015-12-31 | 7.5 | 806 | DB |  | 14 |  |
| vaira | Vaira Ranch, Amador County, California | 38.4133 | −120.9506 | 129 | 2011-10-11 | 2015-12-31 | 15.9 | 583 | GR |  | 8 |  |
| vancouverisland | University of British Columbia Douglas-fir Chronosequence (HDF88), Vancouver Island, B | 49.5364 | −124.9017 | 173 | 2011-11-25 | 2015-12-31 | 8.8 | 1512 | EN |  | 1 | Research at the Young Douglas Fir Flux Station is supported as part of the UBC Integrative laboratory for atmospheric research on greenhouse gas exchange, a Canada Foundation for Innovation Grant, logistical support from Island Timberlands LP and Agrium Inc |
| willowcreek | Willow Creek, Chequamegon-Nicolet National Forest, Wisconsin | 45.8060 | −90.0791 | 521 | 2011-10-25 | 2015-12-31 | 3.9 | 820 | DB |  | 4 | Research at the Willow Creek Ameriflux core site is provided by the Dept. Of Energy Office of Science to the ChEAS Cluster |
| windriver | Thornton T. Munger Research Natural Area, Wind River Experimental Forest, Washington | 45.8213 | −121.9521 | 371 | 2010-04-30 | 2015-12-31 | 9.4 | 2565 | EN |  | 1 | Data and logistical support were provided by the US Forest Service Pacific Northwest Research Station and the University of Washington |
| woodshole | Woods Hole Research Center, Falmouth, Massachusetts | 41.5495 | −70.6432 | 10 | 2011-04-14 | 2015-12-31 | 10.0 | 1178 | DB |  | 5 | Logistical support is provided by the Woods Hole Research Center. |
| worcester | Worcester State University, Worcester, Massachusetts | 42.2697 | 71.8428 | 185 | 2013-05-13 | 2015-12-31 | 8.3 | 1174 | DB | NV | 13 |  |
